# Mice with cisplatin and oxaliplatin-induced painful neuropathy develop distinct early responses to thermal stimuli

**DOI:** 10.1186/1744-8069-5-9

**Published:** 2009-02-26

**Authors:** Lauren E Ta, Philip A Low, Anthony J Windebank

**Affiliations:** 1 Program in Molecular Neuroscience, Mayo Graduate School and Cellular Neurobiology Laboratory, Department of Neurology, Mayo Clinic, College of Medicine, Rochester, MN 55905, USA

## Abstract

**Background:**

Cisplatin has been in use for 40 years for treatment of germ line and other forms of cancer. Oxaliplatin is approved for treatment of metastatic colorectal cancer. Thirty to forty percent of cancer patients receiving these agents develop pain and sensory loss. Oxaliplatin induces distinctive cold-associated dysesthesias in up to 80% of patients.

**Results:**

We have established mouse models of cisplatin and oxaliplatin-induced neuropathy using doses similar to those used in patients. Adult male C57BL6J mice were treated with daily intraperitoneal injection for 5 days, followed by 5 days of rest, for two cycles. Total cumulative doses of 23 mg/kg cisplatin and 30 mg/kg oxaliplatin were used. Behavioral evaluations included cold plate, von Frey, radiant heat, tail immersion, grip strength and exploratory behavior at baseline and at weekly intervals for 8 weeks. Following two treatment cycles, mice in the cisplatin and oxaliplatin treatment groups demonstrated significant mechanical allodynia compared to control mice. In addition, the cisplatin group exhibited significant thermal hyperalgesia in hind paws and tail, and the oxaliplatin group developed significant cold hyperalgesia in hind paws.

**Conclusion:**

We have therefore established a model of platinum drug-induced painful peripheral neuropathy that reflects the differences in early thermal pain responses that are observed in patients treated with either cisplatin or oxaliplatin. This model should be useful in studying the molecular basis for these different pain responses and in designing protective therapeutic strategies.

## Background

Cisplatin has had a central role in cancer chemotherapy for the last 40 years [1,2] and continues to be among the most widely-used antineoplastic drugs in clinical use [3,4]. Cisplatin displays therapeutic efficacy in a broad range of solid tumors especially against testicular, ovarian, and bladder cancers [5,6]. It exerts its antitumor activity by binding to DNA and distorting the helical structure in a way that inhibits transcription [7] and induces apoptotic cell death through DNA damage recognition pathways [8,9]. Peripheral neuropathy remains the most common dose limiting toxicity of cisplatin and limits its clinical use [10,11]. Currently, no effective treatment is available to prevent or treat chemotherapy-induced neuropathy [12].

Significant cisplatin neurotoxicity occurs when cumulative dosage exceeds 300 mg/m^2 ^to 400 mg/m^2 ^[13-16], and is predominantly characterized by sensory neuropathy with initial complaints of pain and paresthesiae in the distal extremities [17,18]. This sensory neuropathy may be delayed in onset, appearing weeks after initiation of therapy. In advanced stages it may progress to severe neuropathic pain and sensory ataxia. In nerve conduction studies, the sensory loss is usually associated with reduction in the amplitude of sensory nerve action potentials, reflecting axonal degeneration [17-21]. Long term cancer survivors may experience residual pain that lasts for many years [22].

Oxaliplatin is a third-generation diaminocyclohexane (DACH) platinum drug that produces cold induced dysesthesia in 90% of patients. The drug is effective for metastatic colorectal cancer [23-25], as well as ovarian and pancreatic cancers [26,27]. Like cisplatin, it is known to cross-link DNA. It is thought to have greater cytotoxic effects than cisplatin because of the bulkier DACH-platinum-adducts [28,29]. Clinically, oxaliplatin induces acute and chronic peripheral neuropathy [30,31]. In contrast to cisplatin, oxaliplatin induces acute neuropathy consisting of cold-induced paraesthesia, dysesthesia or pain in the upper limbs, face, and perioral regions. Symptoms develop early in treatment, typically during the first or second cycle and occur regularly with each cycle of treatment. Similar to cisplatin, about 20 to 30% of patients develop chronic neuropathy with oxaliplatin treatment after longer therapy due to a cumulative toxicity, resulting in pain and loss of sensation.

Although there are distinct differences in the features of the pain associated with platinum compounds, no study has directly compared cisplatin and oxaliplatin in a single animal model. We sought to establish a mouse model that would resemble painful peripheral neuropathy in humans and would be valuable for future elucidation of pain mechanisms associated with cisplatin and oxaliplatin-induced neuropathy. The aim of this study was to determine whether differences exist between cisplatin and oxaliplatin-induced painful neuropathy in mice.

## Results

### Evaluation of general toxicity

All mice survived until the end of study with the exception of one in the cisplatin group that died suddenly at the end of week four. There was no evidence of severe general toxicity. In general, there was no alteration of body temperature and no deterioration in general health status of cis- or oxaliplatin-treated mice compared to control mice.

**Figure 1:**
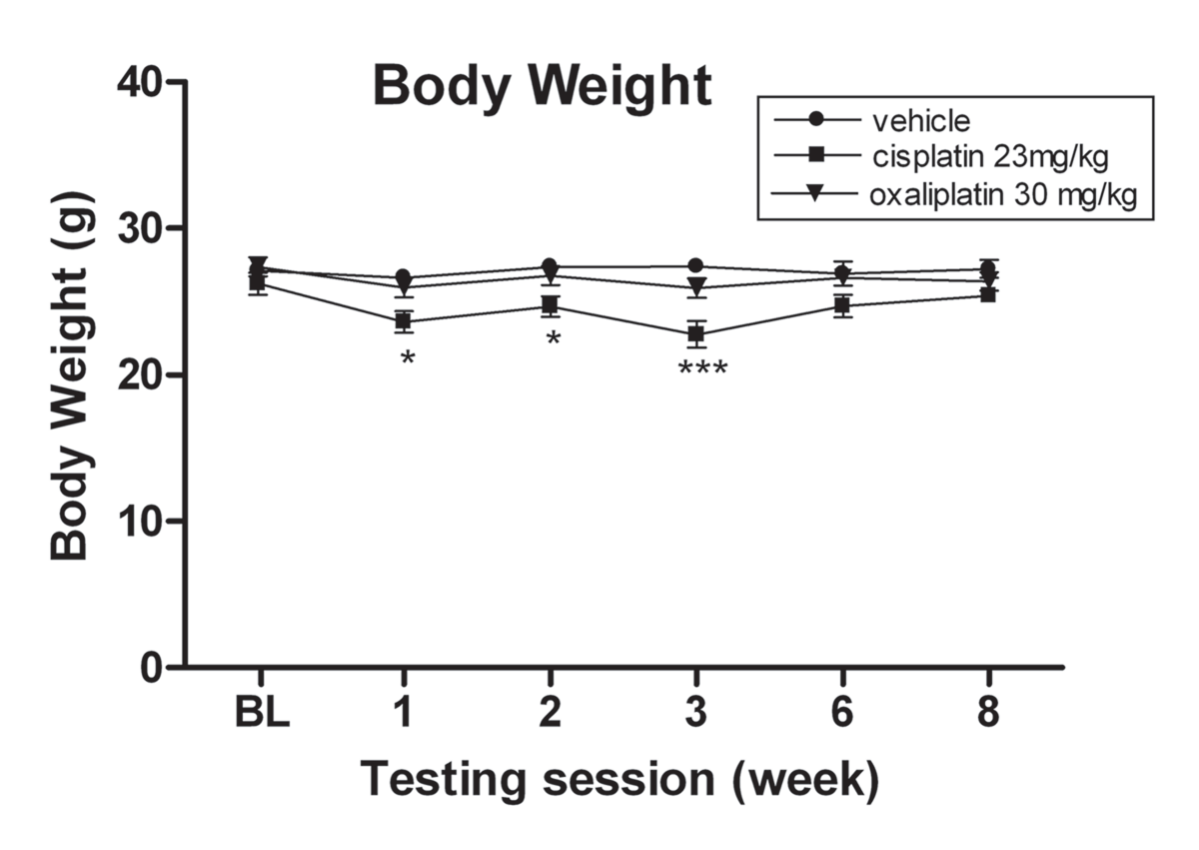
**Cisplatin-treated mice have reduced body weight after each drug treatment cycle**. Data represent the mean ± S.EM of 7 mice, *P < 0.05; ***P < 0.001, two-way ANOVA followed with post hoc analysis.

All mice maintained grooming habits, had normal skin texture on all paws and tail, and exhibited no signs of autotomy. No significant difference of body weight was observed at base line between mice in the three groups (Fig. 1, *P *> 0.05, two-way ANOVA). However, there was a significant decrease in mean body weight in the cisplatin group compared to vehicle control after the first treatment cycle (23.62 ± 0.73 g vs 26.66 ± 0. 49 g, (*P < 0.05, ANOVA); and after the second treatment cycle (22.76 ± 0.91 g vs 27.43 ± 0. 50 g, ***P < 0.001, ANOVA). While a similar trend of weight reduction was seen in the oxaliplatin group after each treatment cycle, this difference was not statistically significant compared to the controls (*P *> 0.05, ANOVA). Mice in both drug groups gradually regained body weight and no significant difference in mean body weight was present between groups after six and eight weeks (Fig. 1, *P *> 0.05, ANOVA).

We observed no evidence of nephrotoxicity following platinum drug treatment. In baseline measurement, prior to drug treatment, blood urea nitrogen (BUN) values for mice in the cisplatin, oxaliplatin, and control treatment groups: 20 mg/dl, 24 mg/dl, and 24 mg/dl respectively. After the 3-week treatment period BUN values were normal for cisplatin, oxaliplatin, and control treatment groups; 27 mg/dl, 15 mg/dl, and 24 mg/dl respectively.

### Cis- and oxaliplatin-treated mice exhibit less exploratory behavior following each treatment cycle

In various pain modality assays, significant differences were observed in the paw lift response in mice following platinum drug treatment. To rule out the possibility that such variances were due to differences in overall activity following drug treatment, we monitored mouse exploratory activity by measuring the horizontal distance traveled in 30 min using Versamax Animal Activity Monitors. Treatment with either cisplatin or oxaliplatin induced significant attenuating effects in the levels of locomotor activity in all mice but was much greater for cisplatin than oxaliplatin.

**Figure 2:**
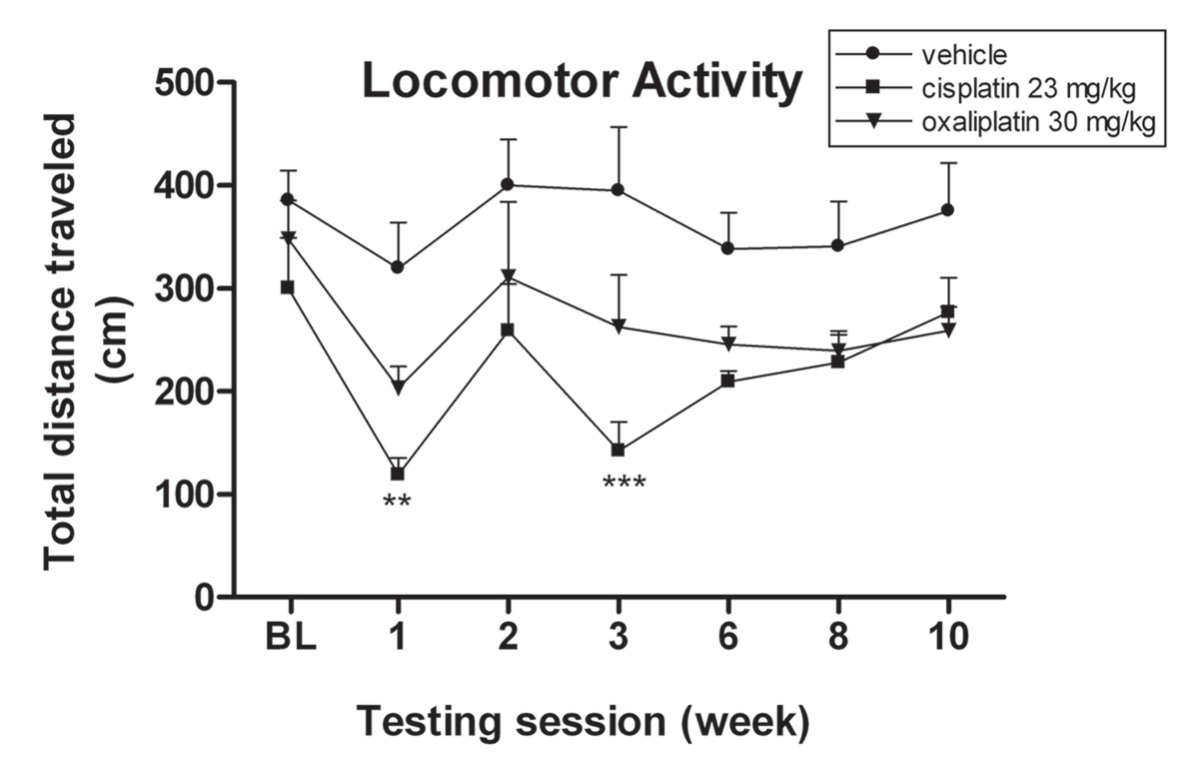
**Cisplatin-treated mice show reduced exploratory activity following each drug treatment cycle**. Data represent the mean ± S.EM of 6 mice, **P < 0.01; ***P < 0.001, two-way ANOVA followed with post hoc analysis.

At baseline, there was no significant difference observed in the mean locomotor activity or horizontal distance traveled between mice for all groups (Fig. 2, *P *> 0.05, two-way ANOVA). However, there was a significant decrease in mean distance traveled by mice in the cisplatin group compared to vehicle controls after the first treatment cycle (119.52 ± 15.88 cm vs 319.92 ± 43.89 cm, (*P < 0.05, ANOVA), and after the second treatment cycle (142.30 ± 28.10 cm vs 394.85 ± 61.93 cm, **P < 0.01, ANOVA). A lesser reduction in mean distance traveled was also observed after each treatment cycle in the oxaliplatin group, but the decrease was not statistically significant (*P *> 0.05, ANOVA). Mice in both drug groups gradually returned to their normal exploratory behaviors and no significant difference was observed in the mean horizontal distance traveled in all groups at weeks six or eight (*P *> 0.05, ANOVA). These data suggest that the observed increases in responses to painful stimuli in the drug treatment groups are not due to an overall increase in general activity.

### Cis- and oxaliplatin-treated mice maintain normal muscle strength

There was no effect of either drug on grip strength which mimics the situation in platinum-treated patients who develop a pure sensory neuropathy. At base line, no significant difference of grip strength was observed between mice in the three groups (*P *> 0.05, two-way ANOVA). Mean baseline grip strength for cisplatin, oxaliplatin, and vehicle groups were 40.40 ± 1.84 g, 41.37 ± 1.49 g, and 41.13 ± 2.62 g, respectively. Cisplatin and oxaliplatin-treated mice maintained similar muscle strength compared to the controls throughout treatment (*P *> 0.05, ANOVA). At week three, mean grip strength for cisplatin, oxaliplatin, and vehicle groups were 39.62 ± 2.28 g, 41.68 ± 1.84 g, and 42.41 ± 2.10 g, respectively. At week six, mean grip strength for cisplatin, oxaliplatin, and vehicle groups were 39.83 ± 2.40 g, 41.00 ± 1.74 g, and 41.28 ± 1.76 g, respectively. At week eight, mean grip strength for cisplatin, oxaliplatin, and vehicle groups were 41.44 ± 1.60 g, 42.07 ± 1.23 g, and 40.03 ± 2.02 g, respectively.

### Oxaliplatin-treated mice develop cold hyperalgesia in the hind paw

**Figure 3:**
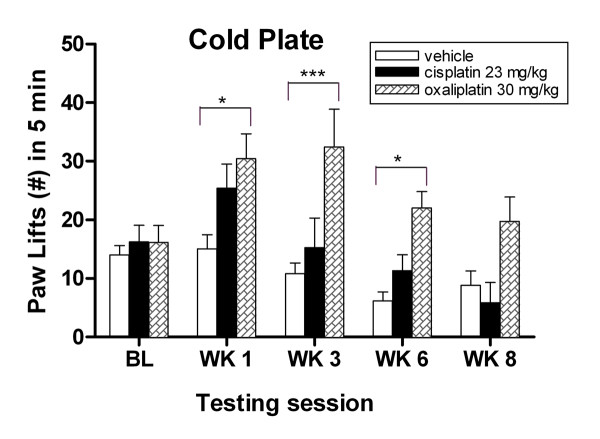
**Oxaliplatin-treated mice exhibit cold hyperalgesia to cold plate assay**. Oxaliplatin-treated mice show increased number of paw lifts to -4.2°C noxious cold stimulus at weeks 1, 3 and 6. Data represent the mean ± S.EM of 7 mice, *P < 0.05; ***P < 0.001, two-way ANOVA followed with post hoc analysis.

Cold pain is a common feature of acute oxaliplatin neuropathy, which was readily detected by the cold plate assay. At baseline, there was no significant difference in the response to cold stimulus at -4.2°C between groups, (Fig. 3, *P *> 0.05, two-way ANOVA). After the first treatment cycle, oxaliplatin -treated mice had a significant (2-fold) increase in the number of paw lifts compared the vehicle-treated controls (30.45 ± 4.27 vs 15.08 ± 2.41, *P < 0.05, ANOVA), and a similar increase in cold hypersensitivity was observed after the second treatment cycle (32.43 ± 6.45 vs 10.84 ± 1.80, ***P < 0.001, two-way ANOVA). This cold hyperalgesia persisted to six weeks (22.00 ± 2.80 vs 6.20 ± 1.50, *P < 0.05, two-way ANOVA) and gradually returned to baseline at week eight (Fig. 3, *P *> 0.05, two-way ANOVA). Although there were more paw lifts to cold stimuli in the cisplatin group, this difference was not statistically significant compared to controls (Fig. 3. *P *> 0.05, two-way ANOVA). The variability of cold hyperalgesia between week 6 and 8 illustrated the acute neurotoxic effect of oxaliplatin drug treatment. Behavioral evidence for the early onset of cold hypersensitivity was also reported in rats, which were given to either low or high doses of oxaliplatin [32]. With sustained use of oxaliplatin drug treatment, neurotoxic effects could manifest as hypoalgesia, a characteristic pain symptoms seen in patient, however, this remains to be examined in mice.

### Cis- and oxaliplatin-treated mice exhibit mechanical allodynia in the hind paw

**Figure 4:**
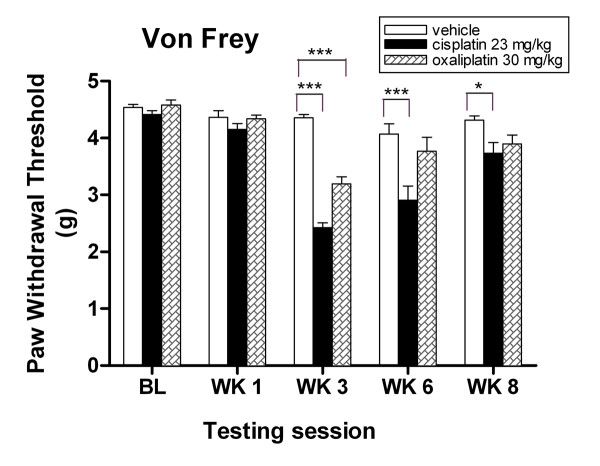
**Cis- and oxaliplatin-treated mice develop mechanical allodynia to von Frey filament assay**. Cis- and oxaliplatin-treated mice show decreased response thresholds to punctate mechanical stimuli at weeks 3, 6, and 8. Data represent the mean ± S.EM of 7 mice, *P < 0.05; ***P < 0.001, two-way ANOVA followed with post hoc analysis.

Platinum drug treatment consistently evoked marked mechanical allodynia, although enhanced mechanical nociception was greater for cisplatin than for oxaliplatin-treated mice (Fig. 4). At baseline, there was no significant difference in response to punctuate mechanical stimuli (von Frey assay) between groups (Fig. 4, *P *> 0.05, two-way ANOVA). However, after completion of two treatment cycles, both cisplatin and oxaliplatin groups exhibited significant 45% and 25% decreases in paw withdrawal thresholds compared to the vehicle group 2.43 ± 0.09 g and 3.19 ± 0.12 g, versus 4.36 ± 0.56 g, respectively (Fig. 4, ***P < 0.001 and ***P < 0.001, two-way ANOVA). While the cisplatin group continued to show significant 28% and 14% decreases in paw withdrawal thresholds compared to the vehicle group at weeks six and eight, respectively (***P < 0.001 and *P < 0.05, two-way ANOVA), however, no statistically significant change was observed with the oxaliplatin group (*P *> 0.05, two-way ANOVA). These findings are consistent with current observations where the neurotoxicity appeared to be less severe with oxaliplatin treatment.

### Cisplatin-treated mice exhibit increased sensitivity to thermal stimuli in the hind paw and the tail

**Figure 5:**
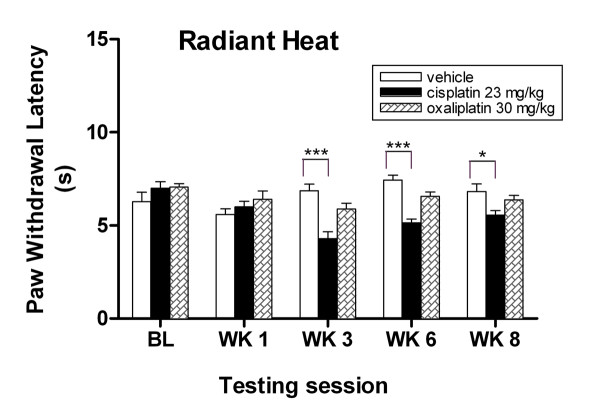
**Cisplatin-treated mice exhibit thermal hyperalgesia to radiant heat assay**. Cisplatin-treated mice show decreased response withdrawal latencies to noxious thermal stimuli at weeks 3, 6, and 8. Data represent the mean ± S.EM of 7 mice, *P < 0.05; ***P < 0.001, two-way ANOVA followed with post hoc analysis.

Clear and robust heat evoked responses were observed in two behavioral heat assays (radiant heat and tail immersion) from cisplatin-treated mice demonstrating that cisplatin induces thermal hyperalgesia in this model. In contrast, no such heat responses were observed for oxaliplatin-treated mice. Cisplatin induced significant thermal nociception to hind paw (Fig. 5), which paralleled with the mechanical nociception as previously observed in Von Frey assay (Fig. 4). At baseline, there was no significant difference between groups in paw withdrawal responses to noxious heat stimuli (Fig. 5, *P *> 0.05, two-way ANOVA). After two cycles of drug treatment, the cisplatin group developed a significant 37% decrease in paw withdrawal latencies compared to the vehicle group (4.29 ± 0.36 s vs 6.85 ± 0. 0.36 s, ***P < 0.001, two-way ANOVA). The cisplatin group continued to exhibit significant 30% and 18% decreases in paw withdrawal latencies compared to the vehicle group at weeks six and eight, respectively (***P < 0.001 and *P < 0.05, two-way ANOVA). Although there was a decrease in paw withdrawal latency in the oxaliplatin group compared to the vehicle group, the difference was not significant after 3, 6 or 8 weeks (*P *> 0.05, two-way ANOVA).

**Figure 6:**
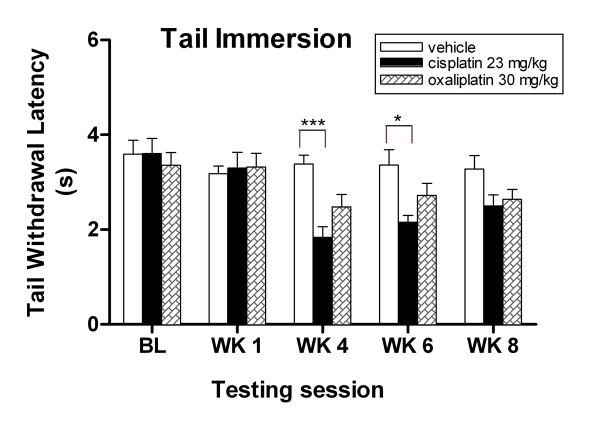
**Cisplatin-treated mice developed thermal hyperagesia to tail immersion assay**. Cisplatin-treated mice show decreased response tail flick latencies to noxious thermal stimuli at weeks 4 and 6. Data represent the mean ± S.EM of 7 mice, *P < 0.05; ***P < 0.001, two-way ANOVA followed with post hoc analysis.

In the tail immersion assay, there was no significant difference in response to noxious heat stimuli between treatment groups at baseline (Fig. 6, *P *> 0.05, ANOVA). However, after two cycles of drug administration, the cisplatin group exhibited highly significant thermal hyperalgesia compared to the vehicle group, and this heat hypersensitivity persisted with a 46% decrease in tail flick latency at week four (1.83 ± 0.23 s vs 3.39 ± 0.19 s, ***P < 0.05, ANOVA), and a 36% decrease in tail flick latency at week six (2.15 ± 0.14 s vs 3.36 ± 0.33 s, *P < 0.05, ANOVA). The lower level of thermal hyperalgesia starting at week 8 depicted the gradual return to baseline of heat response for both paws (Fig. 5) and tail (Fig. 6) in cisplatin treated mice, which indicated the threshold cisplatin dose used in our study. This recovery of responsiveness to heat stimuli may be unlikely to occur with repeated cisplatin administration or higher accumulative doses. Similar observations were found in rats which were given a range of low to high doses of cisplatin [33]. A different pattern of heat evoked response was observed with oxaliplatin. Although there was a decrease in tail flick latency in the oxaliplatin group compared to the vehicle group, the difference was not significant at weeks six and eight (*P *> 0.05, ANOVA).

## Discussion

Patients treated with the platinum compounds cisplatin and oxaliplatin develop dose-related sensory changes. Pain is prominent for both, but cold-induced allodynia or hyperalgesia is a distinctive feature of oxaliplatin treatment [22]. In the present study, we demonstrated that after two treatment cycles of cisplatin and oxaliplatin in mice, different patterns of hyperalgesia emerged for the two drug treatments. Cisplatin-treated mice developed chronic neuropathy with heat hyperalgesia and mechanical allodynia. In contrast, oxaliplatin-treated mice only exhibited acute neuropathy with cold hyperalgesia and mechanical allodynia. These results establish the mouse as a reliable and objective model that can be used to quantitatively assess neuropathic pain behaviors associated with platinum-based drugs.

The intraperitoneal injection of 23 mg/kg cisplatin and 30 mg/kg oxaliplatin approximates human therapeutic doses relative to body weight. In these experiments, treatment doses of cisplatin and oxaliplatin had comparable effects in rodent cell culture models [34]. With these doses, we were able to detect nociceptive responses without causing major deterioration in the general health of the mice. Our previous studies have demonstrated that higher doses of cisplatin result in renal failure and decreased survival [35]. Both platinum drugs also had similar temporal effects on the exploratory activity and body weight of the mice. These were rapidly reversed after cessation of treatment. These observations also confirmed that the increased behavioral responses to cold or heat stimuli could not be explained by an overall increase in locomotor activity.

Since cold pain is one of the hallmark symptoms of oxaliplatin acute neurotoxicity, we also studied the response to cold stimuli using a cold plate assay. Cold plate testing with a range of temperature from -5°C to 15°C has been used to study cold allodynia and hyperalgesia in laboratory rats [36,37]. We found that the oxaliplatin-treated mice exhibited increased responses in the cold plate test at -4.2°C shortly after the first drug cycle. A more robust response was observed after the second drug cycle at week 3. Residual cold hyperalgesia lasted two weeks after the last dose of oxaliplatin. This cold induced hyperalgesia mimics the acute, painful dysesthesia reported by more than 80% of patients treated with oxaliplatin [38]. While some of the mice in the cisplatin group also developed increased cold hypersensitivity, these changes were not significant. Similar to our findings, Ling et al found that after a total dose of 18 mg/kg oxaliplatin administered intravenously over four and half weeks, rats developed cold hyperalgesia to tail immersion at 4°C and mechanical allodynia in the von Frey assay [39]. After a single intraperitoneal injection, these authors found that rats did not develop heat hypersensitivity at 46°C (tail immersion test), but developed cold hypersensitivity at 4°C and 10°C, respectively [32].

We observed that hyperalgesia to thermal stimuli persisted for up to five weeks post treatment for the cisplatin-treated mice. This heat response was consistent in two different thermal stimulus assays. We also observed that the pattern of onset and progression of the heat hyperalgesia was similar to the mechanical allodynia in cisplatin-treated mice, which reflected the consistency of the radiant heat and von Frey assays. Although oxaliplatin-treated mice showed a slight decrease in heat pain threshold, this reduction was not statistically significant. However, they responded significantly to punctate mechanical and cold stimuli; this transient behavioral alteration appeared to be similar to responses frequently observed in oxaliplatin-treated patients.

While there is some agreement about altered nociceptive responses to heat stimuli following chronic cisplatin treatment in animal models [33], there have been some inconsistencies. Many factors could influence pain behavioral outcomes including different species, genetic strain, gender, and type of pain assay [40]. The use of rats and different strains of mice, higher drug dose, and varying heat intensities from previous studies might contribute to different results [41-44].

The chemotherapeutic mechanisms of platinum agents are still not fully understood. Similarly, the reasons for the selective neurotoxicity of platinum compounds and the distinctive, reversible cold sensitivity induced by oxaliplatin are not fully understood. Sensory neurons may be more vulnerable to platinum drugs because they are not protected by the blood brain barrier. We have shown that DRG neurons accumulate high levels of Pt-DNA adducts in a time-dependent manner following cis- and oxaliplatin exposure [34,45]. The amount of platinum binding to DNA is comparable to or exceeds levels known to induce toxic effect to cancer cells. This platinum binding has been shown to induce neurons to enter the cell cycle and undergo apoptosis [46].

Oxaliplatin is known to cause greater toxic effects with less platinum-DNA binding in cancer cells [47-50]. We found a consistently lower level of Pt-DNA adducts in oxaliplatin treated DRG cells; although it was still ten times higher than levels bound to tumor cell DNA [45]. The lower levels of Pt-DNA adduct correlated with less cell death induced by oxaliplatin [34]. These findings are consistent with current observations where the neurotoxicity appeared to be less severe with oxaliplatin treatment, resulting in only transient mechanical and cold hypersensitivity. While the mechanism responsible for this acute neuropathy remains unclear, functional alterations of voltage-gated Na+ [51,52] and glutathione *S*-transferase polymorphisms have also been associated with this oxaliplatin neuropathy [53].

Cisplatin and oxaliplatin treatment have not been shown to induce neuronal loss in peripheral nerves in rodent models [54-57]. This absence of cell death is reflected in the reversibility of nociceptive behaviors seen in treated mice. Animals with experimentally induced neuropathies are frequently seen to have little overt morphological evidence of damage to their peripheral nerves [54-57]. Cisplatin treatment has been shown to result in minimal axonal degeneration or minimal loss of epidermal fibers in mice [42]. In this study, cisplatin and oxaliplatin-treated mice exhibited no significant loss of fiber density in intraepidermal nerve testing compared to controls (data not shown). However, sensory impairment is known to associate with decrease in nerve conduction velocity in animal studies after cisplatin [41,42,44,58] and oxaliplatin drug treatments [56,59].

Changes in neuropeptide expression have been demonstrated in rodent models. Substance P and Calcitonin Gene Related Peptide in pain fibers have been shown to be altered following cis- and oxaliplatin treatment [59,60]. These observed cellular changes could lead to increased activity and hypersensitivity of nociceptive neurons, which may contribute to the spontaneous pain and pain hypersensitivity that are characteristic of neuropathic pain. Future studies dissecting the mechanisms by which these changes are activated could provide further understanding of the pathophysiological mechanism of platinum-induced painful neuropathy.

## Conclusion

The present study suggests that mice may be an excellent animal model in which to examine the role of platinum drugs, and their neurotoxic effects on peripheral nervous systems involved in the induction of pain. These mouse models may also be useful in examining potential therapeutic approaches in treating painful neuropathy associated with platinum drugs in humans.

## Methods

This study was conducted with the approval of the Mayo Clinic Animal Care and Use Committee, and is in compliance with regulations of the National Institutes of Health and ethical guidelines of the International Association for the Study of Pain [61]

### Experimental animals and drugs treatment

Male C57BL6J mice 9–10 weeks of age were obtained from Jackson Lab (Maine). Mice were ordered at least two weeks prior to study to allow for acclimatization. Mice were housed four to a cage in an enriched environment that included a carton hut, bedding blocks, and wood chewing blocks. Mice were closely monitored daily for signs of fighting and aggression during the initial week after arrival. Fighting often occurs in group housing of male mice that are not littermates due to dominance hierarchies. Once the dominant mouse was identified and caged individually, the remaining mice were housed together without signs of fighting. This approach has been shown to be both humane and minimizes injury and aggression [62]. More importantly, it also avoids stress-induced analgesia that could confound basal nociceptive sensitivity [63]. Following two weeks of habituation to the colony, mice were trained with various behavioral testing apparatus for an additional week prior to entering to the study. Mice were 13–14 weeks old and weight was 24–26 g, when the study began. All mice had free access to water and food and were exposed to a standard light cycle of 12 h on and 12 h off.

Oxaliplatin (Sigma-Aldrich, St. Louis, MO) was dissolved in sterile distilled water (1 mg/ml) and prepared each day. Pharmaceutical grade cisplatin (1 mg/ml) was obtained from Bristol-Myers Squibb Company (Princeton, NJ). After habituation to the test environment and baseline measurements of pain sensitivity, mice were randomized to three treatment groups of either cisplatin (2.3 mg/kg), oxaliplatin (3.0 mg/kg), or vehicle (0.9% saline). Treatment doses were based on preliminary dose-escalation studies to determine the threshold concentration producing a measurable difference in pain response between drug-treated and naïve mice (data not shown). Mice were treated with daily intraperitoneal (i.p.) injection for 5 days, followed by 5 days of rest, for two cycles. Total cumulative doses of 23 mg/kg cisplatin and 30 mg/kg oxaliplatin over a total of ten injections were used. These doses and dosing schedules are comparable to patient treatment regimens. A total of 200 μl solution was used for each injection, oxaliplatin solution was prepared with 5% dextrose (Baxter Healthcare, Deerfield, IL), and cisplatin solution was prepared with 0.9% saline (Baxter Healthcare, Deerfield, IL). Injections were performed between the hours of 0900 and 1100.

### Evaluation of general toxicity

Mouse body weight was determined at baseline, before each drug administration and every week up to 8 weeks. In addition, mice were also examined daily for evaluation of general health including observation for signs of hair loss, piloerection, general gait weakness, condition of the hind paws and tail skin, and gastrointestinal disorders.

Ear cavity temperature was measured using an infrared thermometer (model IRT303HACCP, National Product, MD) at baseline and after weeks 1, 3, 6, and 8 prior to performing the behavioral tests. Core body temperature was measured using a rectal probe (Thermalert TH-5 and TCAT-1A Controller, Physitemp Instruments, Inc) at baseline and after weeks 1, 3, and 6 in two mice from each drug treatment group after brief anesthesia with isoflurane.

The nephrotoxicity of cisplatin and oxaliplatin was assessed by blood urea nitrogen (BUN) levels in samples collected at the end of the 3-week drug treatment. Based on normal mouse BUN values (8–33 mg/dl according to normal reference laboratory values from Research Animal Resources at the University of Minnesota-; values as reported for normal untreated C57BL/6 mice [64]), BUN levels > 40 mg/dL were used as an indication of developing nephrotoxicity.

### Behavioral testing

The testing protocol is summarized in Table 1. The order of testing was designed to insure that the least stressful test was done first and to minimize the influence of one test on the next test. After a 2-week acclimation, the mice were trained five times over one week with the specific training protocols to familiarize them with testing procedures prior to entering the study. For the von Frey and paw radiant heat test, mice were allowed to run freely in the apparatus for 20 min before beginning the test. For the tail immersion test, each mouse was loosely wrapped in a partially rolled, moist paper towel for one min; the practice episode was repeated for 3–4 times. For the grip strength test, each mouse was guided for 5 min to pull the trapeze of the grip strength meter 3–4 times. No training was used for the cold plate apparatus. All behavioral tests were carried out in groups of 4–6 experimental mice at baseline, after completion of each drug treatment cycle at weeks 1 and 3, and follow up evaluation at weeks 6 and 8. All behavioral tests were conducted at room temperature (25°C) and between the hour of 0900 and 1600 by one experimenter who was blinded to the drug treatment condition.

**Table 1:** Order and time sequence of testing sessions

**Testing Session**	**Treatment i.p**.	**Activity Assay**	**Grip Strength**	**Cold Plate**	**Von Frey**	**Radiant Heat**	**Tail Immersion**
**Habituation Week**			X		X	X	X

**Baseline**	X	X	X	X	X	X	X

**Week 1**	daily i.p. injection	X	X	X	X	X	X

**Week 2**	5 days rest	X	X				

**Week 3**	daily i.p. injection	X	X	X	X	X	

**Week 4**							X

**Week 6**		X	X	X	X	X	X

**Week 8**		X	X	X	X	X	X

**Week 10**		X					

### Activity monitoring

Monitoring of locomotor activity was carried out at baseline, during drug treatment at weeks 1, 2, 3, and after treatment at weeks 6, 8, and 10 using VersaMax Animal Activity Monitors (AccuScan Model RXYZCM-16, Columbus, OH). The activities of six mice were simultaneously evaluated in six individual open chambers. Mice were allowed to run freely for 5 min prior to behavioral recording for 20 min in the open chamber made of a Plexiglas box (42 × 42 × 30 cm) with wood chip bedding. The VersaMax monitor has infrared sensors located every 2.54 cm along the perimeter (16 infrared beams along each side) and 2.5 cm above the floor. Although the VersaMax monitor collects information in 21 behavioral categories, we only analyzed distance traveled collected in 1-min intervals, collapsed into 10 2-min blocks, averaged and presented as group means ± SEM Data were analyzed by a VersaMax Analyzer (AccuScan Model CDA-8, Columbus, OH).

### Grip strength test

Grip strength was measured using a grip strength meter (Stoelting, Wood Dale, Il) as previously described [65]. The grip strength meter consists of a force transducer with digital display and a metal plate with a trapeze. Each mouse was placed on the plate and was pulled by its tail with increasing force until it was unable to grasp the trapeze and the grip was broken. The instrument digitally captures and displays the peak pull-force achieved. Muscle strength was defined as the peak weight (g) indicated on the display. The value was determined individually as the mean of three trials and presented as group mean ± SEM

### Cold plate assay

Temperatures ranging from -5°C to 4°C at 1°C intervals were used to examine the threshold for cold hyperalgesia in mice. We found that measuring latency to first paw lift was difficult and highly variable. Greater reproducibility was obtained when counting the number of paw lifts in a defined period. When temperature ranged from 0°C to 4°C, naive mice showed few paw lifts (data not shown). However, when the temperature was lowered from -2°C to -5°C, the number of paw lifts for naïve mice increased (data not shown). In preliminary dosing studies, oxaliplatin-treated mice developed significant cold hyperalgesia between -5°C and -3°C, (data not shown). Temperature setting at -4.2°C was used for cold plate testing since there was no tissue damage and the least variability in responses was observed at this temperature (compared with -3°C). The final conditions used were with the Peltier-cooled cold plate preset at -4.2° ± 0.2°C. A temperature sensor was placed directly on the surface of the metal plate to insure accurate temperature reading (TECA, Chicago, IL). For each cold testing session, mice were brought to the testing room and allowed to acclimate for 10 min prior to being individually placed onto the cold metal surface enclosed within a clear plexiglass barrier of 8 cm W × 14 cm D × 14 cm H with a top cover. To insure the accuracy of paw lift counting, we videotaped each cold plate testing session using a video camcorder (Sony DCR-PC1000) and replayed in slow motion. The total number of brisk lifts of either hind paw or jumping was counted as the response to cold hyperalgesia. Movements associated with locomotion were distinct, involving coordinated movement of all four limbs and these were excluded. Mice were only tested once on any given test day to avoid any possible anesthetic or tissue damage effects that could be produced by repeated exposure to a cold surface. A maximum cut off time of 5 min was used to prevent tissue damage. Three separate trials were carried out on three separate days at base line and two separate trials during and after drug treatment at weeks 1, 3, 6, and 8 were averaged and presented as the mean number of paw lifts.

### Von Frey filament assay

For the assessment of mechanical allodynia, an Ugo Basile Dynamic Plantar Aesthesiometer (Stoelting, Wood Dale, Il) using the von Frey filament principle was used [66]. Mice were placed under clear plastic boxes above a wire mesh floor that allowed full access to the paws. Acclimation and exploratory behavior were observed for up to two h until mice became calm and close to motionless. The operator then placed the touch stimulator apparatus under each mouse's hind paw and positioned the calibrated metal filament below the target area of the hind paw. After pressing the start key, an electrodynamic actuator of the apparatus lifts the metal filament (diameter of 0.5 mm). The filament touches the plantar surface and exerts a continuous vertical force of 0 to 5 g over a 10 sec interval until the hind paw withdraws and activates a stop signal. The instrument automatically registered the weight intensity threshold in g that triggered paw withdrawal. Each hind paw was tested alternately with an interval of 5 min for four trials. Paw movement associated with locomotion or weight shifting was not counted as withdrawal responses. Paw withdrawal threshold of eight trials from both hind paws of each mouse were averaged and recorded as mean ± SEM.

### Radiant heat assay

For the assessment of thermal hyperalgesia, a Hargreaves' test [67] was conducted using a Plantar Ugo Basile apparatus (Stoelting, Wood Dale, Il). Mice can move freely in this apparatus on an elevated glass surface with plastic boxes above as the top cover. Mice are given a two h acclimation period prior to testing until they become calm and motionless. A calibrated infrared light source of high intensity was applied perpendicular on the plantar surface of each mouse's hind paw. The rising temperature on the bottom of the hind paw caused the mouse to move its paw; the change in paw position alters reflected light and stops the timer. Latency to paw withdrawal was automatically recorded for each trial. If the mouse did not withdraw its hind paw within 15 s, the testing trial terminated to prevent tissue damage and 15 s was recorded. Each hind paw was tested alternately with an interval of 5 min for four trials. Paw withdrawal latency of eight trials from both hind paws of each mouse was averaged and recorded as mean ± SEM.

### Tail immersion assay

For assessment of tail thermal hyperalgesia, a tail immersion test was conducted as previously described with modifications [68]. For each testing session, mice were brought to the testing room and each was individually acclimatized three to four times in a moist paper towel for a minute duration without tail immersion. Next, each mouse was swiftly and gently wrapped in a slightly moist paper towel and held in the investigator's hand with minimal restraint to allow the distal one-third of the tail to be immersed in a water bath. The water bath was preset at 50.5° ± 0.5°C and the temperature was verified with an independent extra temperature sensor. Only when the mouse was calm and its tail was relaxed, was the next step of tail immersion pursued. If the mouse became agitated, it was unwrapped and the testing protocol restarted. Latency to vigorous tail flick was recorded during three trials separated by at least 30 min, and three trials were averaged and presented as mean ± SEM. Cutoff time was set at 20 s, after which mouse was removed regardless of behavioral response.

### Statistical analyses

All data are expressed as mean ± standard error of the mean (SEM). Results were illustrated and analyzed using Graph pad Prism version 4 (Graphpad Software, San Diego, USA). Statistical analyses were performed using two-way ANOVA with drug treatments and time as independent variables to examine for the differences in responses across treatment groups. Follow-up analysis was conducted using the Bonferroni test. A *P *value of less than 0.05 is considered as statistically significant.

## Competing interests

The authors declare that they have no competing interests.

## Authors' contributions

LET designed the study, conducted the behavioral studies, performed the data analysis, and prepared the final manuscript. PAL participated in the design of the study and interpretation of the results. AJW is the senior investigator for the project and was actively participated in the study design, interpretation of the results, and the final manuscript. All authors have read and approved the final manuscript.
